# SARS-CoV-2 Nsp2 Contributes to Inflammation by Activating NF-κB

**DOI:** 10.3390/v15020334

**Published:** 2023-01-24

**Authors:** Émile Lacasse, Leslie Gudimard, Isabelle Dubuc, Annie Gravel, Isabelle Allaeys, Éric Boilard, Louis Flamand

**Affiliations:** 1 Axe Maladies Infectieuses et Immunitaires, Centre de Recherche du Centre Hospitalier Universitaire de Québec-Université Laval, Québec City, QC G1V 4G2, Canada; 2 Département de Microbiologie-Infectiologie et d’Immunologie, Université Laval, Québec City, QC G1V 0A6, Canada

**Keywords:** SARS-CoV-2, inflammation, non-structural protein 2

## Abstract

COVID-19 is associated with robust inflammation and partially impaired antiviral responses. The modulation of inflammatory gene expression by SARS-CoV-2 is not completely understood. In this study, we characterized the inflammatory and antiviral responses mounted during SARS-CoV-2 infection. K18-hACE2 mice were infected with a Wuhan-like strain of SARS-CoV-2, and the transcriptional and translational expression interferons (IFNs), cytokines, and chemokines were analyzed in mouse lung homogenates. Our results show that the infection of mice with SARS-CoV-2 induces the expression of several pro-inflammatory CC and CXC chemokines activated through NF-κB but weakly IL1β and IL18 whose expression are more characteristic of inflammasome formation. We also observed the downregulation of several inflammasome effectors. The modulation of innate response, following expressions of non-structural protein 2 (Nsp2) and SARS-CoV-2 infection, was assessed by measuring IFNβ expression and NF-κB modulation in human pulmonary cells. A robust activation of the NF-κB p65 subunit was induced following the infection of human cells with the corresponding NF-κB-driven inflammatory signature. We identified that Nsp2 expression induced the activation of the IFNβ promoter through its NF-κB regulatory domain as well as activation of p65 subunit phosphorylation. The present studies suggest that SARS-CoV-2 skews the antiviral response in favor of an NF-κB-driven inflammatory response, a hallmark of acute COVID-19 and for which Nsp2 should be considered an important contributor.

## 1. Introduction

Severe acute respiratory syndrome coronavirus 2 (SARS-CoV-2) emerged in Wuhan, China, in December 2019 leading to the coronavirus infectious disease 2019 (COVID-19) global outbreak [1]. Severe acute respiratory syndrome coronavirus 2 is a member of the Coronaviridae family, Orthocoronavirinae subfamily, Betacoronaviruses genus, Sarbecovirus subgenus [2]. The SARS-CoV-2 genome consists of a single-stranded (ssRNA) positive RNA genome of an approximate length of 29.7 kb [2,3]. The SARS-CoV-2 genome encodes 4 structural proteins (spike (S), envelope (E), membrane (M), and nucleocapsid (N)), 7 accessory proteins (ORF3a, ORF6, ORF7a, ORF7b, ORF8, and ORF10) and ORF1ab, a large open reading frame (ORF) that encodes a large polyprotein that gets cleaved in 16 non-structural proteins (Nsp1-16) [2]. The SARS-CoV-2 infection requires the binding of the S protein to the human Angiotensin-Converting Enzyme 2 (ACE2) followed by the cleavage of the S2 subunit by transmembrane protease serine protease-2 (TMPRSS-2) and ADAM metallopeptidase domain 17 (ADAM17) [4,5]. Finally, SARS-CoV-2 enters its host cell by endocytosis [6].

One of the first host defense mechanisms against pathogens such as viruses is the innate immune response that is initiated by the recognition of pathogen-associated molecular patterns (PAMPs) by cellular sensors [7]. One of the main effector systems triggered in response to viral infections is interferon (IFN) production [8]. In the case of infection by viruses like SARS-CoV-2, the type I IFN pathways can be activated by two different processes. One of them involves the recognition of double-stranded RNA (dsRNA) SARS-CoV-2 replication intermediates by RIG-I-like receptors (RLRs) such as retinoic acid-inducible gene I (RIG-I) and/or melanoma differentiation gene 5 (MDA5) sensors located in the cytoplasm [9,10]. Viral RNA recognition by these sensors leads to the phosphorylation, dimerization, and nuclear translocation of IFN regulatory factor 3 (IRF3) and IRF7. In parallel, NF-κB activation is initiated and together with IRF3/7, *IFNB*1 gene transcription is initiated [11,12,13]. Type I IFN transcription can also be activated by the recognition of dsRNA by the Toll-like receptor 3 (TLR3) or by the recognition of ssRNA by TLR7/8 [14]. Toll-like receptor activation results in *IFNB*1 gene transcription through similar signaling pathways [15].

The products of type I *IFN* genes, IFNα/β, are secreted in the extracellular space. The IFN receptor (IFNAR1-2) engagement activates the Janus kinases signal transducer and activator of the transcription proteins (JAK-STAT) pathway that leads to the expression of several dozen interferon-stimulated genes (*ISG*s) whose products are responsible for establishing the antiviral defense [16,17,18].

In addition to activating IFN signaling, the recognition of viral PAMPs, as well as damage-associated molecular patterns (DAMPs) generated by viral replication by TLRs, NOD-like Receptors (NLRs) or AIM2-like receptors (ALR), leads to the activation of NF-κB-responsive genes and the formation of the inflammasome. These pathways can initiate the production and activation of several pro-inflammatory mediators such as cytokines and chemokines [19,20]. Relevant to SARS-CoV-2 infection, severe forms of COVID-19 are fueled by a hyperinflammatory reaction culminating in acute respiratory distress (ARD) [21,22,23].

Unsurprisingly, various components of the type I IFN response are targeted by many viruses and other pathogens. As was observed with SARS-CoV, viral proteins such as Nsp1 can target signaling proteins and modulate the immune response of the host [24,25]. Furthermore, more recent studies indicate that SARS-CoV-2 can evade the type I IFN response by inducing translational shutdown mediated by Nsp1 and Nsp2 [26,27,28]. Conversely, SARS-CoV-2 Nsp2 seems to amplify the type I IFN response [27,29,30]. Most studies conducted on type I IFN response evasion by SARS-CoV-2 were carried out using single protein expression systems that cannot fully recapitulate infection or conditions where several viral and cellular proteins are expressed simultaneously. Conversely, some studies suggest that SARS-CoV-2 induces type I IFN expression and a robust ISGs expression [29,30]. Moreover, SARS-CoV and SARS-CoV-2 Nsp2 appear to have characteristics distinguishing these pandemic viruses from Bat-SARS-like CoV [31]. In the current study, we used K18-hACE2 mice infected with the Wuhan-like SARS-CoV-2 strain and a human pulmonary epithelial cell line to characterize innate immune response modulation during infection. We also examined whether Nsp2 impacted IFN production and whether this effect was conserved during the co-expression of Nsp1, the main type I IFN antagonist produced concomitantly with Nsp2 [26,32,33]. Our results indicated that SARS-CoV-2 downregulated the IFNβ at a protein level in mice and human cells meanwhile inducing a robust CC and CXC chemokine response in mice. Moreover, our work provides evidence that Nsp2 is not an IFNβ1 antagonist and activates the IFNβ1 promoter through the activation of the NF-κB pathway. Our results suggest that SARS-CoV-2 skews the antiviral response in favor of an NF-κB-driven inflammatory response for which Nsp2 should be considered an important contributor.

## 2. Materials and Methods

Cell culture and virus. The HEK293T and Vero cells were purchased from American Type Culture Collection (Manassas, VA, USA), A549-hACE2 cells were obtained from Biodefense and Emerging Infections Research Resources Repository (BEI Resources, Manassas, VA, USA). These cell lines were passaged twice a week. The HEK293T and A549 cells were cultured in Dulbecco’s Modified Eagle Medium (DMEM) (Corning Cellgro, Manassas, VA, USA) with 10% fetal bovine serum (FBS) (Corning Cellgro), 10 mM HEPES pH 7.2, 1% (*v*/*v*) nonessential amino acid (Multicell Wisent Inc., St-Bruno, QC, Canada), and 5 μg/mL of Plasmocin^®^ (Invivogen, San Diego, CA, USA) to prevent mycoplasma contamination. Vero cells were cultured in Medium 199 (Multicell Wisent Inc., St-Bruno, QC, Canada) supplemented with 10% FBS and 5 μg/mL of Plasmocin^®^. Cell lines were grown at 37 °C with 5% CO_2_. Sendai virus (SeV) was obtained from Charles River Laboratory (Saint-Constant, QC, Canada) and SARS-CoV-2 Wuhan-like strain (LSPQ, B1 lineage) from the Laboratoire de Santé Publique du Québec ((LSPQ) Sainte-Anne-de-Bellevue, QC, Canada); this strain will be considered as a wild-type strain. SARS-CoV-2 was propagated on Vero cells and the supernatant of infected cells was used for infection experiments. The infectious titer of viral preparations was 1.8 × 10^6^ Tissue Culture Infectious Dose_50_/mL (TCID_50_/mL) for mouse experiments and 5.24 × 10^6^ TCID_50_/mL for in vitro experiments. The A549-hACE2 were infected at a multiplicity of infection (MOI) of 1 for 1 h, then washed twice with phosphate-buffered saline 1X (PBS) followed by addition of culture media. Experiments involving infectious SARS-CoV-2 viruses were performed in a BSL-3 facility.

Determination of the viral titer. Vero cells were plated in a 96-well plate (2 × 10^4^/well) and infected with 200 µL of serially diluted viral preparations or lung homogenates in M199 media supplemented with 10 mM HEPES pH 7.2, 1 mM of sodium pyruvate, 2.5 g/L of glucose, 5 μg/mL Plasmocin^®^, and 2% FBS. Three days post-infection plates were analyzed for signs of cytophathic effects using a EVOS M5000 microscope (ThermoFisher Scientific, Waltham, MA, USA) and the viral titer was determined using the Karber method [34].

Mice. The B6.Cg-Tg(K18-hACE2)2Prlmn/J (stock#3034860) mice were purchased from the Jackson Laboratories (Bar Harbor, ME, USA). Nine-week-old male and female mice were infected with 25 µL of saline containing 9 × 10^3^ (TCID_50_/mL) of SARS-CoV-2 or 25 µL of saline for mock-infected mice. Mouse weight was recorded every day until euthanasia. Mice were sacrificed on day 3 post-infection and lungs were collected for RNA extraction and tissue homogenization for cytokine and infectious titer (TCID_50_/mL) analysis.

Plasmids and reagents. SARS-CoV-2 non-structural protein (Nsp) 1 and 2 expression vectors were generated by amplifying the genes from SARS-CoV-2 RNA. Nsp1 and 2 genes were cloned into pENTR (L1-L2) using Hifi DNA Assembly (New England Biolabs, Ipswich, MA, USA). The LR Recombination Gateway (Thermo Fisher Scientific, Waltham, MA, USA) was used to recombine Nsp1 and Nsp2 genes into pCDNA5-TO (obtained from Dr. Anne Claude Gingras, Lunenfeld-Tanenbaum Research Institute, Toronto, ON, Canada). To generate an Nsp1-Nsp2 polyprotein coding vector, Nsp1-P2A [35] was cloned into the pcDNA5-TO-Nsp2 vector using PCR overlap cloning with Hifi DNA Assembly. The Nsp2 codon-optimized vector pDONR223 SARS-CoV-2 Nsp2 (Addgene #141256) was used to generate sleeping beauty inducible vector using Addgene kit 1000000155 [32] by subcloning Nsp2 into pMAGIC(R4-R3) with Hifi DNA Assembly. The SB100 transposase expression vector was obtained from Addgene (#34879), expression vector IFN-β-LUC was obtained from Dr. Nathalie Grandvaux (CHUM, Montreal, QC, Canada), ISRE-LUC (Interferon-sensitive response element) expression vector was obtained from BD Biosciences (Mississauga, ON, Canada), the NF-κB-LUC expression vector was obtained from Michel J. Tremblay (CHUL, Quebec, Qc, Canada), and PRD1-III-LU C vector was obtained from Dr. Tom Maniatis (Zuckerman Institute, New York, NY, USA). Polyinosinic-polycytidylic acid (poly[I:C]) was purchased from Cytiva (Mississauga, ON, Canada). The BCA Protein Assay kit was purchased from (Thermo Fisher Scientific, Waltham, MA, USA). Primers used for plasmid construction are listed in the Supplementary Table S1.

Transfection and stable cell line generation. HEK293T cells were transfected using TransIT^®^-LT1 (Mirus, Madison, WI, USA) reagent with indicated expression vectors. Poly(I:C) transfections were performed using lipofectamine 3000 reagent (ThermoFisher Scientific) at a ratio of 1:5. To generate Nsp2 and GFP inducible cell line, A549-hACE2 (4 × 10^6^ cells) were nucleofected using program X-001 on the Nucleofector^®^ 2b Device (Lonza, Bâle, Switzerland) with 8 µg of DNA mix containing sleeping beauty and the SB100 transposase vector at a molar ratio of 11:1 (vector:transposase). Forty-eight hours post-nucleofection, transfected cells were selected with 1µg/mL of puromycin (Multicell Wisent Inc., St-Bruno, QC, Canada) for at least one week then clones were isolated using a single-cell cloning approach.

Protein expression. HEK293T cells were plated in a 6-well plate (6.5 × 10^5^/well) 24 h before transfection. A549-hACE2 cells cultured in a 6-well plate (2 × 10^5^ cells) were infected with SARS-CoV-2. Forty-eight hours post-transfection, cells were lysed in radio-immunoprecipitation assay (RIPA) buffer with Halt Protease Inhibitor Cocktail or Halt Phosphatase Inhibitor Cocktail (Thermo Fisher Scientific, Waltham, MA, USA) or directly in Laemmli 2X buffer. The proteins were separated by SDS/PAGE gel and transferred to a PVDF low-fluorescence membrane (Bio-Rad Laboratories Ltd., Mississauga, ON, Canada). Membranes were incubated with 1 μg/mL of mouse anti-Flag (Applied Biological Materials Inc., Richmond, BC, Canada), 0.25 μg/mL of rabbit anti-SARS-CoV-2-Nsp1 (Genetex, Irvine, CA, USA), 0.25 μg/mL of rabbit anti-SARS-CoV-2-Nsp2 (Genetex), 5 μg/mL of rabbit anti-SARS-CoV-2-N (Rockland Immunochemicals Inc., Limerick, PA, USA), 1/1000 dilution rabbit anti-NF-κB-p65 (Cell Signaling Technology, Danvers, MA, USA), or 1/1000 dilution rabbit anti-phospho-NF-κB-p65-Ser536 (Cell Signaling Technology) for 1 h at room temperature or 16 h at 4 °C. Peroxidase-labeled goat anti-mouse IgG (Jackson Immunoresearch Laboratories Inc., West Grove, PA, USA) (40 ng/mL) or peroxidase-labeled goat anti-rabbit (Jackson Immunoresearch Laboratories Inc.) (80 ng/mL) were used as secondary antibodies for 1 h at room temperature and revealed with the addition of Clarity or Clarity Max ECL reagent (Bio-Rad Laboratories Ltd.). ChemiDoc MP Imaging System (Bio-Rad Laboratories Ltd.) or radiological films (Mandel, Guelph, ON, Canada) were used to capture images. Rabbit anti-tubulin 2A and 2B (Abcam Inc., Toronto, ON, Canada) (0.66 μg/mL) or Stain-Free Imaging Technology^®^ (Bio-Rad Laboratories Ltd.) were used as loading controls. Band volumes for quantitative Western Blot were analyzed and normalized using Image Lab Software (Bio-Rad Laboratories Ltd.). All immunoblots were performed at least two times and one representative image is shown.

Reporter assays. HEK293T cells were plated in 24-well plates (1.6 × 10^5^/well) and transfected with 50 ng to 100 ng of reporter vectors and 30 ng to 300 ng of Nsp1, Nsp2 or Nsp1-Nsp2 vectors brought to 0.5 μg/well with the empty expression vector. Twenty-four hours post-transfection, transfected cells were infected with 20 hemagglutinin units of SeV or stimulated with 500 units of IFNα (PBL Assay Science, Piscataway, NJ, USA). Sixteen hours later, cells were lysed, and the luciferase activity was determined as previously described [33].

IFNβ induction and ISG induction. HEK293T cells were plated (3 × 10^4^/well) in 12-well plates and transfected with 0.2 μg of Nsp1 vector, 0.6 μg of Nsp2 vector, or 0.8 μg of Nsp1-Nsp2 vector complete to 1 μg/well with empty pCDNA5. Twenty-four hours post-transfection, cells were infected with 40 hemagglutinin units of SeV for sixteen hours. Supernatants and cells were collected separately, and cells were lysed in 0.5 mL of QIAzol reagent (Qiagen, Toronto, ON, Canada). Samples were stored at −80 °C until future analysis.

Infection and poly(I:C) stimulation. A549-hACE2 cells were plated (7.5 × 10^4^/well) in 12-well plates and infected with Wuhan-like SARS-CoV-2 strain following the same procedure described above. Twenty-four hours post-infection, cells were transfected with 2 µg/mL of poly(I:C) for 16 h. Supernatants and cells were collected separately, and cells were lysed in 0.5 mL of QIAzol reagent (Qiagen). Supernatants were incubated with 1% triton for one hour at room temperature to inactivate SARS-CoV-2. Samples were stored at −80 °C until analyzed.

IFNß quantification. The IFNβ in the supernatant was quantified with the Human IFN-beta DuoSet enzyme-linked immunosorbent assay (ELISA) kit, according to the supplier recommendations (R&D Systems Inc., Toronto, ON, Canada).

Multiplex cytokines quantification. Cytokines in mouse lung homogenates were measured using a custom ProcartaPlexTM Mouse Mix & Match Panels kit (Invitrogen, Waltham, MA, USA) on the Bio-Plex 200 (Bio-Rad Laboratories Ltd.).

Quantitative real-time PCR analysis. Total RNA from cell cultures was extracted following QIAzol protocol and RNA from mouse lungs was extracted using the Bead Mill Tissue RNA Purification Kit and the Omni Bead Ruptor Bead Mill homogenizer (Kennesaw, GA). Following extraction, residual DNA was removed by treating the samples with DNAse I (Roche, Mississauga, ON, Canada). For the quantification of human gene expression and mouse Cxcl1, Ccl2, Isg56(Ifit1), Ifny, and Ifna, RNA was reverse transcribed to cDNA using SuperScript™ IV VILO™ mastermix (ThermoFisher Scientific). Quantitative real-time PCR (qPCR) was performed using the SsoAdvanced Universal Probes Supermix (Bio-Rad Laboratories Ltd.) for IFNB1 gene and GAPDH as the housekeeping gene. SsoAdvanced Universal SYBR Green Supermix (Bio-Rad Laboratories Ltd.) was used for human *ISG15* and *ISG56* and mouse genes including *Gapdh* as the housekeeping gene on the Rotor-Gene Q 5plex (Qiagen). The RT-qPCR primers and probes are listed in Supplementary Table S2.

Digital PCR analysis. SARS-CoV-2 viral RNA loads were determined using Droplet Digital PCR (ddPCR) supermix for probes without dUTP (Bio-Rad Laboratories Ltd.) and the QX200 Droplet Digital PCR System Workflow (Bio-Rad Laboratories Ltd.). The ddPCR primers and probes are listed in the Supplementary Table S2.

RT^2^ profiler PCR Arrays. The RNA extracted from mouse lungs as described above was cleaned up using On-Column DNAse using RNAse-Free DNase Set (Qiagen) and RNeasy Mini Kit (Qiagen). The RNA was reverse transcribed using RT2 First Strand Kit (Qiagen). qPCR and quality control were performed using RT^2^ SYBR^®^ Green ROC FAST Mastermix (Qiagen) and RT^2^ profiler PCR Arrays: Mouse Antiviral response (Qiagen). Data analyses were performed using the GeneGlobe (Qiagen) analyzing tool. Genes of the RT^2^ profiler PCR Arrays are listed in the Supplementary Table S3.

Immunofluorescence. A549-hACE2 cells were plated (1.6 × 10^4^/well) in 8-well chamber slides. Twenty-four hours later, cells were infected with SARS-CoV-2 as described above. Forty-eight hours post-infection, cells were fixed in 2% paraformaldehyde in PBS for one hour at room temperature. Cells were then incubated for thirty minutes in blocking solution (PBS with 0.1% bovine serum albumin (BSA), 3% FBS, 0.1% Triton X-100, and 1 mM EDTA) then with 17 μg/mL of rabbit anti-SARS-CoV-2-N (Rockland Immunochemicals Inc., Limerick, PA, USA) in the blocking solution for one hour at room temperature. After, cells were washed three times for 5 min with PBS and incubated with 4 μg/mL of goat anti-rabbit-Alexa-488 (Thermo Fisher Scientific) in the blocking solution for 30 min at room temperature. Finally, cells were washed for 5 min in PBS and incubated into PBS 1X with 1.67 µg/mL of DAPI (Invivogen). Cells were washed for 5 min in PBS, mounted with ProLong Diamond Antifade reagent (Thermo Fisher Scientific) and images were acquired using a Z2 confocal microscope with LSM 800 scanning system (Zeiss, Germany). Images were captured with a 20x objective (Zeiss, Apochromat). ZEN 2.3 software (Zeiss) was used to acquire and process images. Z-stack projections of 3 μm in total thickness are represented. Contrast adjustments and image processing were performed using Zen lite (Zeiss).

## 3. Results

**Figure 1:**
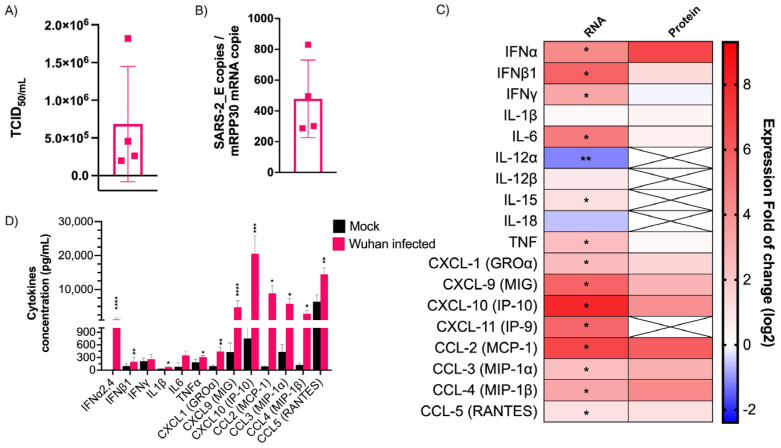
Cytokine mRNA and protein expression profile following infection of K18-ACE2 mice with Wuhan strain. Infected or mock mouse lung tissues were collected three days post-infection (*n* = 4/group). (**A**) The SARS-CoV-2 *E* gene copy number was evaluated by ddPCR using lung RNA and expressed as number per copy of *Rpp30* mRNA (mean ± SD). (**B**) Infectious viral titers were determined in lung homogenates and expressed in TCID_50/mL_ (mean ± SD). (**C**) Gene expression was evaluated by RT-qPCR and cytokine concentration in lung homogenates determined using a 13-plex Luminex panel. Cytokine gene expression and concentration levels are presented as heatmaps with results expressed as fold (log_2_) relative to mock-infected mice (mean). Statistical analyses were performed by comparing 2^(−ΔCt)^ values for each gene in the control group and infected groups with a nonparametric T-test. (**D**) Cytokine concentrations in lung homogenates. Results are expressed as mean ± SD. For protein quantification, statistical analyses were performed by comparing the normalized concentration for each cytokine in the control group and infected groups with a nonparametric *t*-test. * *p* < 0.05, ** *p* < 0.01, *** *p* < 0.001, **** *p* < 0.0001.

SARS-CoV-2 induces robust CC and CXC chemokine production but limited type I IFN production in mice. To study the innate immune response developed during infection, K18-hACE2 mice were infected with the Wuhan-like strain SARS-CoV-2. Three days post-infection, the mice were euthanized and their lungs used for further analyses. No change in body weight or temperature was observed during this time [36]. As shown in Figure 1A,B, all the infected mice had significant pulmonary infectious viral loads and expressed abundant SARS-CoV-2 E gene copy numbers. When the antiviral-related gene expression was measured, Chemokine C-X-C Ligand 10 (*Cxcl10* [*Ip-10*]), Chemokine C-C Ligand 2 (*Ccl2* [*Mcp-1*]), *Cxcl11* (*Ip-9*), *Cxcl9* (*Mig*), *Ifnb1*, and Interleukin 6 (*Il-6*) were the main cytokine genes upregulated (Figure 1C). *Ifnγ*, Tumor necrosis factor α (*Tnfα*, *Cxcl1(Groa*), *Ifnα, Ccl3* (*Mip-1α), Ccl4* (*Mip-1β)* genes were also upregulated, but to a lesser extent. In contrast, *Il12α* and *Il18* genes were downregulated or poorly induced. When analyzed at the protein level, CC and CXC chemokines were efficiently produced in response to infection. In contrast, despite robust *Ifnβ1, Ifnγ, Il-6*, and *Tnf* gene expression, few gene products were measured (Figure 1C). In terms of relative abundance, chemokines were the mediators produced at the greatest levels, being several hundred to a thousand times more abundant than other pro-inflammatory cytokines and type I IFN (Figure 1D). These results show that SARS-CoV-2 triggers an innate immune response in mice dominated by chemokines.

**Figure 2:**
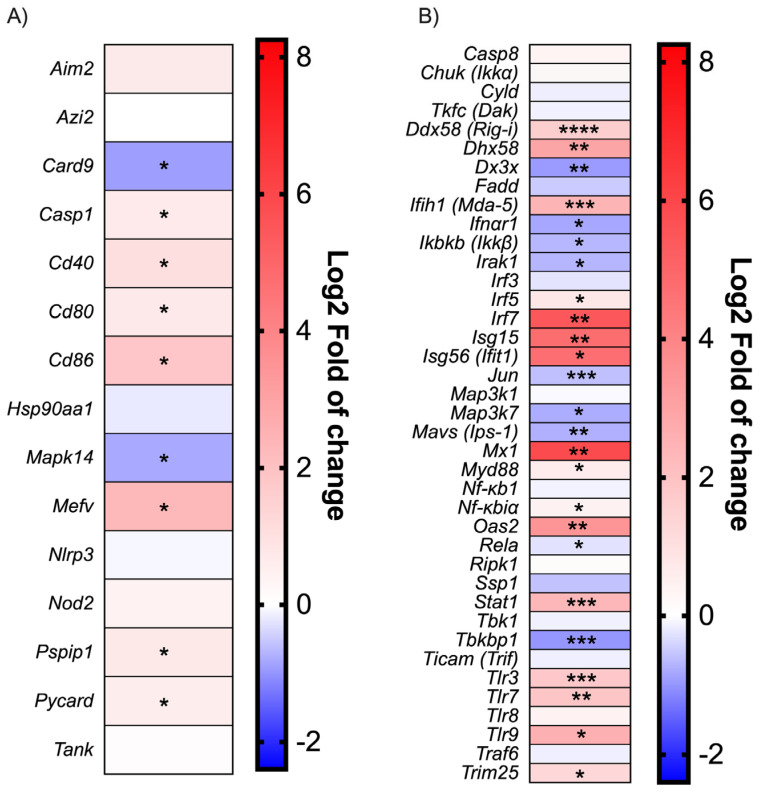
Antiviral response gene expression following infection with Wuhan strain. Heat map representation of cytokines and inflammatory-related genes (**A**) and Type I IFN production and signalization-related genes (**B**). Results are expressed as fold (log_2_) relative to mock-infected mice (mean, *n* = 4/group). For gene expression, statistical analyses were performed by comparing 2^(−ΔCt)^ values for each gene in the control group and infected groups with a nonparametric *t*-test. * *p* < 0.05, ** *p* < 0.01, *** *p* < 0.001, **** *p* < 0.0001.

SARS-CoV-2 infection in mice does not induce an inflammatory reaction mediated by the inflammasome. Along with the cytokines studied above, the expression of several genes involved in the Toll-like receptor (TLR), NOD-like receptor (NLR) and RIG-like receptor (RLR) signaling pathways were measured. Despite the robust upregulation of the Mediterranean fever gene (*Mefv*) implicated in inflammasome formation [37] and proinflammatory cytokine release, inflammasome components such as the Apoptosis-associated speck-like protein containing a CARD (*Pycard*), Proline-serine-threonine phosphatase-interacting protein 1(*Pspip1*), Absent in melanoma 2 (*Aim2*), Caspase 1 (*Casp1*), and NLR family pyrin domain containing 3 (*Nlrp3*) were weakly modulated early in infection (Figure 2A). Moreover, Caspase recruitment domain-containing protein 9 (*Card9*) and Mitogen-activated protein kinase 14 (*Mapk14*), implicated in the inflammatory process, were downregulated. This finding is consistent with the lack of inflammasome-characteristic cytokines such as IL1β and IL18 (Figure 1C).

Modulation of the IFN activation pathways during SARS-CoV-2 infection. As shown in the Figure 2B, many downstream effector genes such as Interleukin-1 receptor-associated kinase 1 (*Irak1*), Transcription factor (*Jun*), *Mavs* [*Ips-1*], Mitogen-activated protein kinase kinase 7 (*Map3k7[Tak1*]) were downregulated during infection. Moreover, Canopy FGF signaling regulator 3 (*Cnpy3*), a TLR chaperon [38], was also downregulated, which could impair the recognition of viral PAMPs. The inhibitor of the nuclear factor kappa-B kinase subunit beta (*Ikbkb[Ikkb]*) was also downregulated; meanwhile, nuclear factor kappa-B1 (*Nf-kb1*) expression was not affected. As IKBKB inhibit the NF-κB complex formation [39], a reduction in IKBKB would result in higher overall NF-kB activity. On the other hand, cytoplasmic and endosomal ssRNA and dsRNA sensors, such as Tlr3, Tlr7, Tlr8, DExD/H-box helicase 58 (*Ddx58*[*Rig-i*)), 2′-5′-oligoadénylate synthetase 2 (Oas2), and Interferon-induced helicase C domain-containing protein 1 (*Ifih1* [*Mda-5*]), were upregulated during SARS-CoV-2 infection (Figure 2B). Lastly, *Irf7* gene transcription was robustly induced following infection (Figure 2B).

Robust ISG expression despite low-level type I IFN production during SARS-CoV-2 infection. The expression of genes associated with type I IFN signaling was monitored during infection (Figure 2B). Interferon-stimulated genes such as *Isg15, Isg56 (IFIT1*), and Interferon-induced GTP-binding protein Mx1 (*Mx1*) were strongly induced following infection. In agreement with those results, Stat1 gene transcription was also upregulated. Conversely, Ifnar1 expression was downregulated.

**Figure 3:**
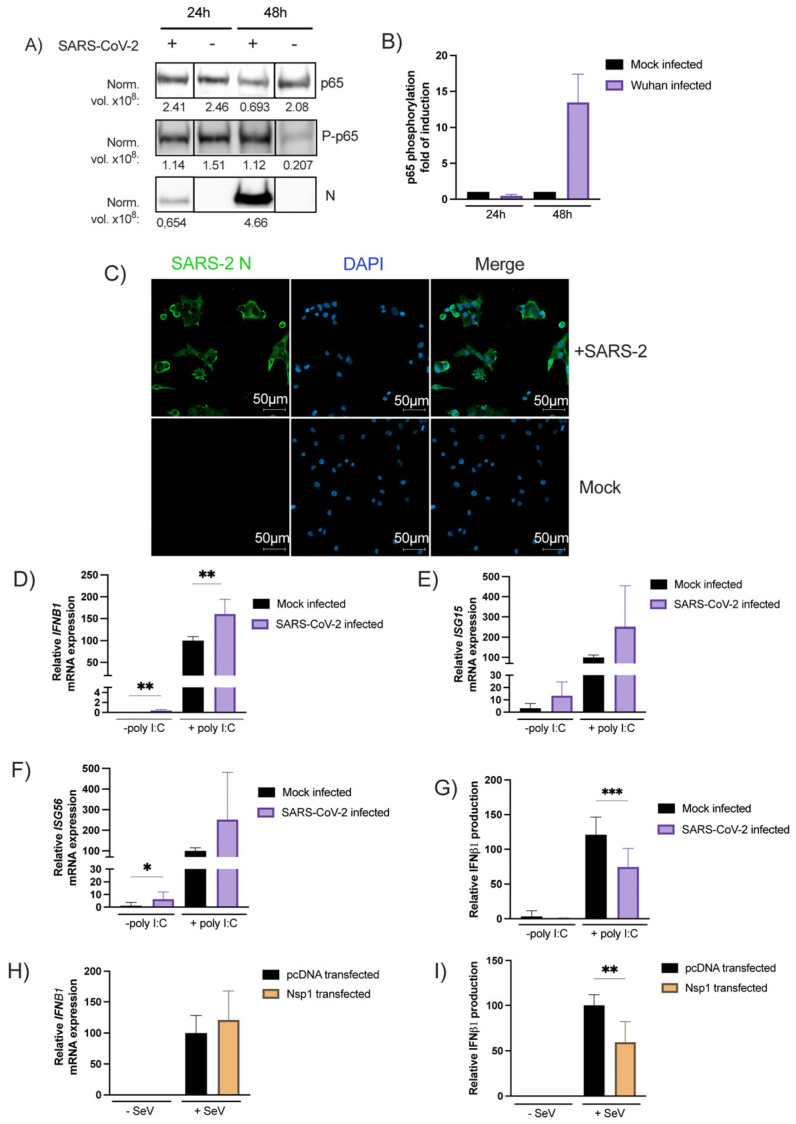
The NF-κB p65 subunit expression and phosphorylation following infection with the SARS-CoV-2 (**A**,**B**). A549-hACE2 cells in 6-well plate infected as described in the method section. Then, harvest RIPA buffer with Halt Phosphatase Inhibitor Cocktail detected by Western Blot with anti-p65, anti-P-p65 or anti-SARS-CoV-2-N and Stain-Free Imaging Technology^®^ as loading control (Supplementary Figure S1) and expressed as normalized levels (Norm. vol. ×10^8^) below the blot. Then, NF-κB p65 phosphorylation is normalized with p65 subunit expression and expressed as fold of induction relative to the mock-infected control (mean ± SD, *n* = 2 replicates/condition) (**B**). SARS-CoV-2 Nucleocapsid staining on A549-hACE2 infected cells. Forty-eight hours post-infection cells were fixed and stained as described in the materials and methods section. This staining was performed a least three times and a representative picture was shown. (**C**). Effect of SARS-CoV-2 infection on poly(I:C) IFNB1 mRNA expression (**D**), ISG15 mRNA (**E**) or ISG56 mRNA expression (**F**), induced IFNβ1 production (**G**). Twenty-four hours post-seeding, A549-hACE2 cells were infected with SARS-CoV-2 as described in the materials and methods section. Thirty-two hours post-infection, SARS-CoV-2 and mock-infected cells were stimulated with poly(I:C) and RNA extracted and analyzed with RT-qPCR while IFNβ1 was measured in the supernatant by ELISA. Effect of Nsp1 on IFNb1 mRNA (H) and protein (**I**) expression. Cells were transfected with expression vectors Nsp1 or an empty vector and infected or not with SeV as IFNβ inducer. RNA was isolated and analyzed for *IFNB1* mRNA (**H**), Supernatants were collected and assayed for IFNβ production (**I**). All experiments were performed twice in triplicate and the compilation of the data is shown. Results are expressed as activation percentage relative to the poly I:C transfected or SeV-infected negative control (mean ± SD (*n* = 6 replicates/condition)). Statistical analyses were performed by comparing mock-infected or pcDNA-transfected control with corresponding condition with a nonparametric T-test. * *p* < 0.05, ** *p* < 0.01, *** *p* < 0.001.

SARS-CoV-2 infection induces NF-κB phosphorylation. To verify the ability of SARS-CoV-2 to activate NF-κB signaling, NF-κB p65 subunit phosphorylation was quantified by Western Blot in infected A549-hACE2 cells. As shown in Figure 3A, at 24 h post- infection the virus did not affect the overall p65 expression while at 48 h post-infection, a three-fold reduction in the p65 expression was noted relative to the mock treated cells. On the other hand, SARS-CoV-2 infection (48 h) induced p65 phosphorylation by more than 4-fold. The p65 phosphorylation increment correlated with the viral load, as determined by N protein expression (Figure 3A). When p65 phosphorylation is normalized with the overall p65 expression levels, a 15-fold induction in p65 phosphorylation is measured 48 h post-infection relative to control cells (Figure 3B).

SARS-CoV-2 induces *IFNB1* transcription, inhibits IFNβ protein synthesis, and does not impair ISG transcription. A549-hACE2 cells were treated with the SARS-CoV-2 Wuhan-like strain and infection confirmed by immunofluorescence using anti-nucleocapsid (N) antibodies (Figure 3C). The *IFNB1, ISG15, ISG56* mRNA levels and IFNβ1 secretion were measured using mock-infected or SARS-CoV-2-infected cells treated or not with poly(I:C), a type I IFN inducer. We made use of poly(I:C) to determine whether SARS-CoV-2 would affect exogenous IFN inducing stimuli. The IFNβ1 mRNA quantification revealed that SARS-CoV-2 infection efficiently induced *IFNB1* gene transcription. Stimulation with poly(I:C) amplified *IFNB1* mRNA expression (Figure 3D). Similar results were obtained for *ISG15* and *ISG56* gene expression ± poly(I:C) (Figure 3E,F). At the protein level, no IFNβ1 was detected in the supernatant of infected cells with SARS-CoV-2 (Figure 3G). The virus was also capable of partially inhibiting the poly(I:C)-induced IFNB1 secretion (Figure 3G). This translational inhibition was recapitulated with Nsp1 transfection (Figure 3H,I).

**Figure 4:**
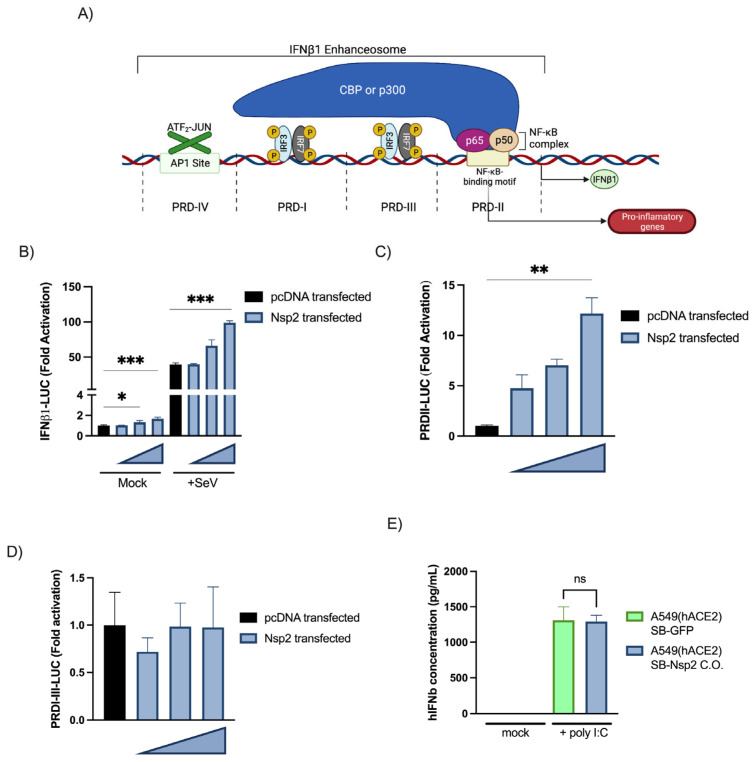
Characterization of IFNβ promoter activation by Nsp2(C-E). Overview of the IFNβ promoter (**A**). Impact of Nsp2 expression on the IFNβ promoter (**B**), the NF-κB responsive elements, positive regulatory domain II (PRDII) (**C**) and IRF3-responsive elements, the positive regulatory domains I and III (PRDI-III) (**D**) of those promoters. Reporter essays were performed as described above with 300 ng, 200 ng, and 100 ng of vector encoding for Nsp2. All experiments were performed twice in triplicate, and one representative experiment is shown. Results are expressed as fold activation relative to the pcDNA mock-infected control (mean ± SD, *n* = 3 replicates/condition). Effect of Nsp2 expression on the IFNβ1 production (**E**). Twenty-four hours post-seeding, A549-hACE2 SB-Nsp2 or SB-GFP cells were induced with 0.5 µg/mL of Doxycycline. Thirty-two hours post-induction, cells were stimulated with poly(I:C) for sixteen hours and IFNβ1 was measured in the supernatant by ELISA. All experiments were performed twice in triplicate and the compilation of the data is shown. Results are expressed as activation percentage relative to the poly I:C mock-infected control (mean ± SD (*n* = 6 replicates/condition)). Statistical analyses were performed by comparing corresponding condition with mock-transfected control with a nonparametric one-way ANOVA with Dunn’s correction. ns: not significant, * *p* < 0.05, ** *p* < 0.01, *** *p* < 0.001.

Characterization of IFNβ1 promoter activation by Nsp2. Th *IFNB* gene is regulated by a complex promoter containing positive regulatory domains I and III (PRD-I-III) (IRF3/7 responsive elements) and a PRD-II (NF-κB-responsive element) (Figure 4A) [40]. As Nsp2′s effects on IFNB1 are somewhat controversial in the literature, we opted to determine whether Nsp2 is a Type I IFN antagonist or not. An expression vector coding for Nsp2 was co-transfected into HEK293T cells together with an IFNβ1 promoter luciferase reporter. As shown in Figure 4B, Nsp2 expression activated the IFNβ1 promoter and amplified the response to SeV infection. [40]. The results with PRD-I-III and PRD-II demonstrated that Nsp2 activated the NF-κB binding domain of the IFNβ1 promoter (Figure 4C) while having no effects on the PRD-I-III elements (Figure 4D). To determine whether Nsp2 could induce IFNβ1 production and secretion, we generated Nsp2- or GFP-inducible A549(hACE2) and measured IFNβ1 release in the supernatant following mock or poly (I:C) stimulation. The results showed that Nsp2 expression did not impair nor induce IFNB1 production in a detectable way (Figure 4E).

**Figure 5:**
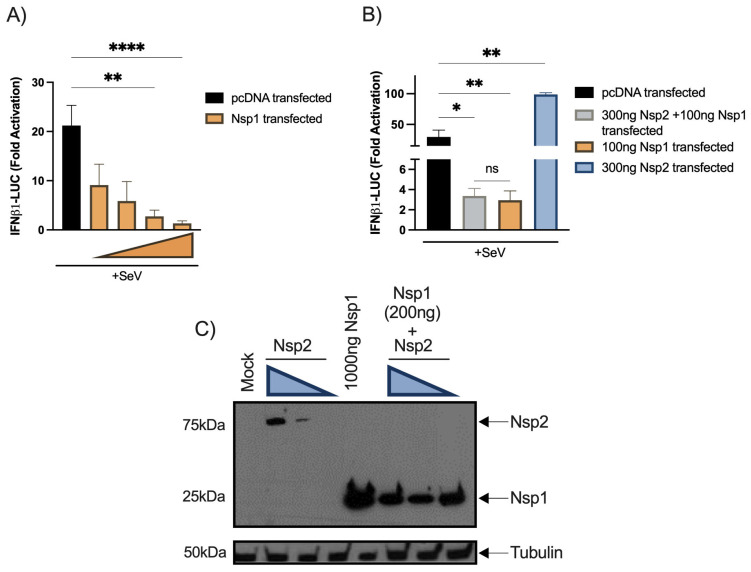
Effect of SARS-CoV-2-Nsp1 and Nsp1-Nsp2 cotransfection on SeV-induced IFNβ1 promoter activation. HEK293T cells were seeded, transfected, and simulated according to the procedure described above and the luciferase activity was measured then standardized as described for other reporter assays. Doses of 300 ng, 100 ng, 30 ng, and 3 ng per well of Nsp1 vectors for Nsp1 alone experimentation (**A**). HEK293T cells were seeded, transfected, and simulated according to the procedure described above and the luciferase activity was measured then standardized as described for other reporter assays. Doses of 300 ng of Nsp2 aand 100 ng of Nsp1 per well were used (**B**). All experiments were performed twice in triplicate and the compilation of the data is shown. Results are expressed as fold activation relative to the pcDNA mock-infected control (mean ± SD, *n* = 6 replicates/condition). The Nsp1-Nsp2 cotransfection and Nsp1 transfection conditions were compared to the mock-transfected control using nonparametric one-way ANOVA with Dunn’s correction and Nsp2 conditions were compared using nonparametric one-way ANOVA. The Nsp1-Nsp2 cotransfection and Nsp1 transfection conditions were compared using nonparametric *t*-test. * *p* < 0.05, ** *p* < 0.01, **** *p* < 0.0001, ns: not significant. Protein expression of Nsp1 and Nsp2 in individual transfection and cotransfection (**C**). HEK293T were seeded in 6-well plate. Twenty-four hours after, cells were transfected with control vector or Nsp1 expression vector or Nsp2 expression vector. Doses of 800 ng, 500 ng, and 100 ng of Nsp2 vector were used. Forty-eight hours post-transfection, cells were lysed in SDS PAGE 2X buffer and detected by Western Blot with an anti-FLAG (viral protein) and rabbit anti-tubulinβ (loading control) on radiological films. The Western Blot was performed two times and one representative experiment is shown.

Nsp2 co-transfection fails to reduce the inhibitory effect of Nsp1. Since Nsp1 was identified as the main type I IFN antagonist by several groups, we aimed at evaluating whether both proteins might antagonize each other. First, Nsp1 the expression vector was co-transfected into HEK293T cells together with the IFNβ1 reporter to validate its inhibitory activity (Figure 5A). Second, the Nsp1 and Nsp2 vectors were cotransfected with IFNβ1 reporter activation. No significant differences between cells co-transfected with Nsp1 and Nsp2 vectors and cells singly transfected with an Nsp1 vector alone were detected (Figure 5B). When transfected cells were analyzed for Nsp1 and Nsp2 expression, Nsp2 could be efficiently detected only in the absence of Nsp1, suggesting that Nsp1 inhibited Nsp2 translation (Figure 5C).

**Figure 6:**
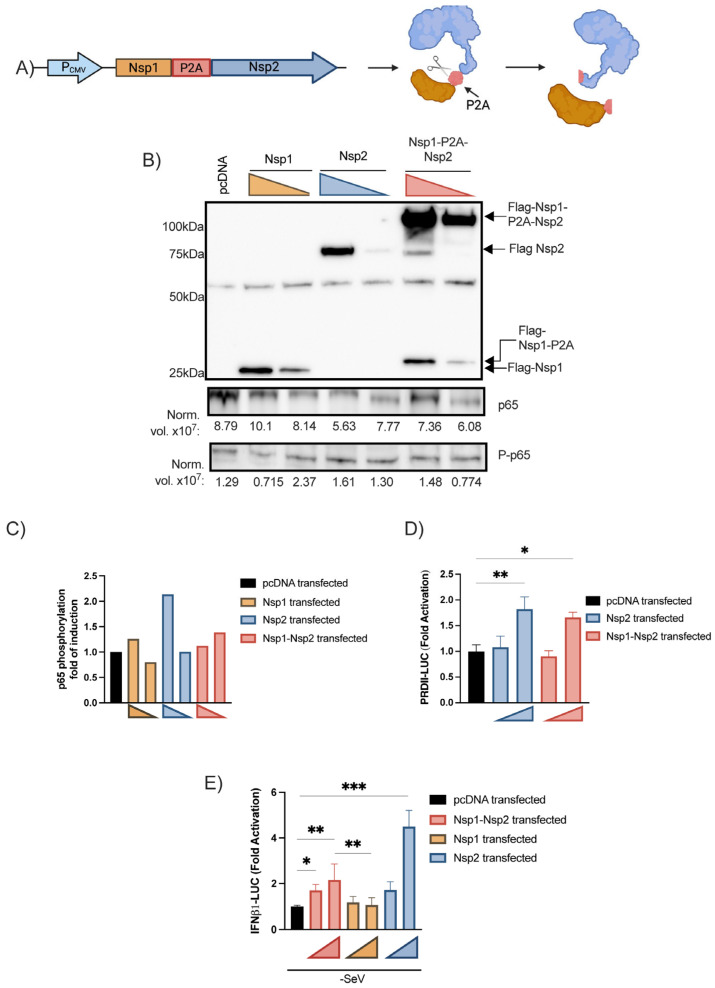
Effects of Nsp1-Nsp2 polyprotein on IFNβ and ISRE-luc promoter activation and p65 subunit phosphorylation. Schematic representation of the Nsp1-P2A-Nsp2 encoding vector cleaved products (**A**). Nsp1 and Nsp2 coexpression and p65 expression following polyprotein vector transfection (**B**) and the relative p65 phosphorylation (**C**). HEK293T were seeded in 6-well plate. Twenty-four hours after, cells were transfected with 0.4 pmol or 0.2 pmol of Nsp1 expression vector, Nsp2 expression or Nsp1-Nsp2 polyprotein vector. Forty-eight hours post-transfection, cells were lysed with Halt Phosphatase Inhibitor Cocktail and detected by Western Blot with anti-FLAG (viral protein), anti-p65, phospho-NF-κB-p65-Ser536, and Stain-Free Imaging Technology^®^ as loading control (Supplementary Figure S2) expressed as normalized levels (Norm. vol. ×10^8^) below the blot. Then, NF-κB p65 phosphorylation was normalized with p65 subunit expression and expressed as fold of induction relative to the mock-infected control. This experiment was performed twice, and one representative experiment is shown. Effects of Nsp1-Nsp2 on PRDII reporter activation, (**D**) IFNβ1-luc, (**E**). IFNβ1 essay was performed as described above with 20 or 80 fmol of vector encoding for Nsp1, Nsp2 or Nsp1-Nsp2 polyprotein. PRDII reporter essays were performed using 40 or 80 fmol of following vectors. All experiments were performed twice in triplicate and the compilation of the data is shown. Results are expressed as fold activation relative to the pcDNA mock-infected control (mean ± SD, *n* = 6 replicates/condition). The Nsp1-Nsp2 and Nsp1 conditions were compared to the mock control using nonparametric one-way ANOVA with Dunn’s correction. The Nsp1-Nsp2 and Nsp1 conditions were compared together using nonparametric T-test. The Nsp2 transfection conditions were compared to the control using nonparametric one-way ANOVA with Dunn’s correction. * *p* < 0.05, ** *p* < 0.01, *** *p* < 0.001.

Effects of Nsp1-Nsp2 polyprotein on IFNβ pathways. To circumvent the fact that Nsp1 prevented the expression of Nsp2, we designed a vector expressing a Nsp1-P2A-Nsp2 polyprotein (schematized in Figure 6A). The polyprotein vector was transfected into HEK293T cells and the polyprotein along with Nsp1 and Nsp2 individual proteins were detected by Western Blot (Figure 6B). The p65 and phosphorylated p65 expression levels were also quantified on the same cell lysate showing that Nsp2 increases the p65 phosphorylation (Figure 6B,C). Next, the polyprotein-encoding vector was co-transfected with PRD-II and IFNβ1 luciferase reporters.

As for Nsp2 alone, Nsp1 and Nsp2 coexpression activated the PRD-II (Figure 6D). Under basal conditions, a significant increase in IFNβ promoter activity was observed in the presence of Nsp1 and Nsp2 (Figure 6E). These results indicate that in the presence of Nsp1, the Nsp2 protein remains capable of activating the IFNβ1 promoter and NF-κB responsive element, suggesting that Nsp2 partially antagonizes Nsp1 effects while favoring the expression of inflammatory genes.

## 4. Discussion

In this study, we demonstrated that SARS-CoV-2 induced robust *Ifnb1* gene transcription during the infection of K18-hACE2 mouse and human cells, but low to even undetectable IFNβ1 protein release. *Ifnb1* gene expression is regulated by the coordinated actions of IRF3 and NF-κB, which are constitutively expressed in most cells [41,42]. Infected cells can therefore respond rapidly to incoming viruses by inducing *Ifnb1* gene expression and IFNβ production even before viruses can deploy their anti-viral defense mechanisms. In the case of SARS-CoV-2, several viral proteins are reported to possess activities that antagonize the innate immune response such as type I IFN production. The most potent SARS-CoV-2 protein antagonizing the IFN response is the Nsp1 protein that induces a global shutdown of cellular mRNA translation [26,27]. The fact that several ISGs, such as *Isg15*, *Igs56*, and *Mx1* are highly upregulated (Figure 2B) during infection suggests that infected cells release sufficient IFNβ to induce the expression of genes associated with antiviral defense mechanisms. However, the establishment of an antiviral state is contingent on efficient ISG mRNA translation. To find out whether the anti-viral state is efficiently put in place, we examined *Irf7* gene expression and IFNα production. IRF7, constitutively expressed in plasmacytoid dendritic cells and B cells and induced in many other cell types by viral infections, is the main transcription factor responsible for the activation of IFNα promoters [12,43]. Plasmacytoid dendritic cells and B cell types do not appear to express ACE2 nor transmembrane serine protease 2 (TMPRSS2) [6], suggesting that they cannot be directly infected by SARS-CoV-2. In response to infection by SARS-CoV-2, *Irf7* is among the genes most highly induced in infected mice (Figure 2B), suggesting that the recognition of the infection by cellular sensors and downstream signaling molecules is functional.

Despite the relatively low type I IFN production and the *Ifnar* downregulation, ISGs were highly expressed in mice following SARS-CoV-2 infection suggesting that ISG activation could also rely on an IFN-independent mechanism. Indeed, it had been shown that the human cytomegalovirus and the vesicular stomatitis virus could induce ISG expression in *Ifnar* KO mice by an IRF1- and an IRF3-dependent manner [44,45,46]. A study from Zhou, Z. et al. [30] also detected a robust ISG expression in the bronchioalveolar lavage fluids (BALFs) of patient with severe COVID-19 but failed to detect type I IFN expression at the RNA level. This contradiction may be due to the time point of the BALF sampling and the kind of sample, BALFs versus lung homogenate.

Banerjee, A. et al. [29] recently reported that SARS-CoV-2 efficiently induced a type I IFN transcriptional response upon the infection of pulmonary epithelial cells. Our work supports similar findings. In contrast, work by others [26,27] clearly shows that this virus can also strongly inhibit the IFNβ1 protein expression. This apparent contradiction can be explained by the fact that certain studies measure RNA expression, while others evaluate protein synthesis. In fact, knowing that SARS-CoV-2 Nsp1 suppresses mRNA translation, the study of both mRNA and protein synthesis is necessary to reach proper conclusions [26,27]. In that regard, our work confirms that the infection of pulmonary epithelial cells by SARS-CoV-2 induced *IFN*β gene expression and even potentiated the response to IFN-inducing agents such as poly(I:C) (Figure 3D). When IFNβ production in the supernatant was assessed however, partial inhibition in IFNβ production was measured only when infection was combined with poly(I:C) stimulation (Figure 3G).

Relative to type I IFNs and interleukins, CC and CXC chemokines were produced at high levels during infection by all SARS-CoV-2 strains in agreement with observations made in the lungs of humans infected with SARS-CoV-2 and suffering from severe COVID-19 [22,30]. In the lungs of patients with severe COVID-19, CXCL8 (IL-8) and CXCL1 (GROα) were the predominant CXC chemokines. Mice do not encode the *Cxcl8* gene, but do have CXCL1, which was produced at high levels (Figure 1C,D). As in humans, CCL2 was the most prominent CC chemokine produced during infection (Figure 1C,D). While CXCL1 and CXCL8 (human) are mainly involved in neutrophil recruitment and activation, CCL2 is the main cytokine implicated in monocyte recruitment as well as TH1 polarization [47,48,49]. Put together, the concerted actions of these chemokines likely lead to a massive recruitment of leukocytes responsible of the ARDS observed in severe COVID-19 case [22,23].

The weak IL-1β production combined with the lack of modulation or downregulation of inflammasome effector and IL-18 suggest that the pathological inflammation following the K18-hACE2 mice did not rely on the inflammasome. This inflammation fueled by the inflammasome has been describe in the works of Zeng, J. et al. [50] and Sefik et al. [51] in AAV-hACE2-transduced mice. This discrepancy may be explained by the difference between the two mouse models. K18-hACE2 mice express the hACE2 receptor and support SARS-CoV-2 infection in a broad range of tissues, while in AAV-hACE2 mice, the expression of the hACE2 receptor is limited to the respiratory tract [52]. This major difference could impact the disease course and may change the inflammatory pattern. Also, in our study we euthanized mice on day 3 before the apparition of clinical symptoms, while other studies have made analyses at later time points.

While our data in infected mice suggest that inflammasome was the inflammation trigger, we show that early infection induces robust NF-κB activation that could drive the expression of chemokines such as CXCL1,9,10,11 or CCL2,3,4,5. Combined with the lack of type I IFN production, in in vitro conditions, this finding suggests that the virus skews the immune response toward an exaggerated inflammatory response rather than an antiviral response, as previously hypothesized [53]. This overwhelming inflammatory response represents a major determinant of the pathogenesis and morbidity observed during COVID-19. In support, the use of dexamethasone, a non-specific anti-inflammatory drug, has proven effective in reducing mortality and the length of hospital stay for patients with COVID-19 requiring oxygen supply [54].

Original to this work, we provided evidence that Nsp2 activates the NF-κB pathway and the IFNβ1 promoter. This effect of Nsp2 could be demonstrated when Nsp1-Nsp2 were generated from a common polyprotein as with the situation observed during infection (Figure 6D,E). When expressed individually, Nsp1 prevented the efficient expression of Nsp2 (Figure 5C).

In summary, the current study reveals that SARS-CoV-2 infection triggers the vigorous expression of antiviral and inflammatory genes. However, in both mouse and cell lines, IFN synthesis is sub-optimal, a consequence of the translational shutdown mediated by Nsp1. The IFN shutdown in infected cells is however incomplete, in part due to the action of other viral proteins such as Nsp2 that partially antagonize the actions of Nsp1. As such, our work highlights the importance of studying viral protein functions in the context of infection. The use of recombinant mutant viruses will be helpful in delineating the synergistic/antagonizing functions of non-structural and accessory proteins during infection. In contrast to IFN, elevated inflammatory gene expression did translate into the production of high levels of several inflammatory chemokines, many of which are regulated by NF-κB. Considering our results demonstrating that Nsp2 activates the NF-κB pathway, Nsp2 should be considered as a potential contributor to the pathogenesis observed during SARS-CoV-2 infection.

## Data Availability

Not applicable.

## Supplementary Materials

The following supporting information can be downloaded at: https://www.mdpi.com/article/10.3390/v15020334/s1.
