# Supplementary material for: SARS-CoV-2 Nsp2 Contributes to Inflammation by Activating NF-κB
Source: Viruses. 2023 Jan 24;15(2):334. doi: 10.3390/v15020334 (PMC9964531; doi:10.3390/v15020334)
Supplement: Supplementary file 1 [file viruses-15-00334-s001.zip › viruses-2142702-supplementary.pdf]

# Suplementary Figure 1

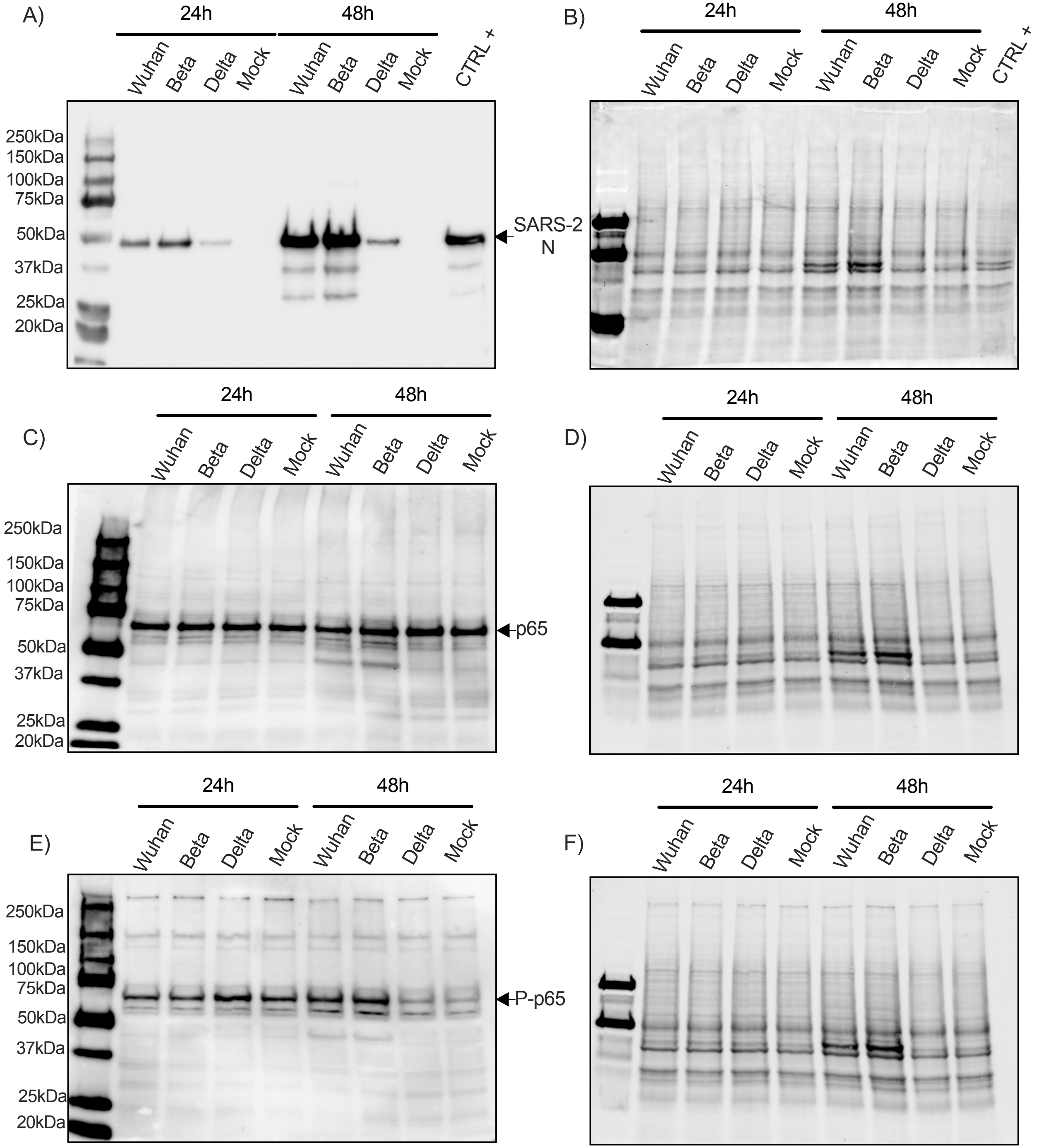

**Figure S1.** Uncropped blots (A, C, D) and Stain-Free blot (B, D, F) corresponding to the relative protein expression level of SARS-CoV-2 N protein (A, B), total p65 subunit (C,D) and phosphorylated p65 (E,F) following 24h or 48h infection with SARS-CoV-2 Whuan-like, Beta or Delta strains.

# Suplementary Figure 2

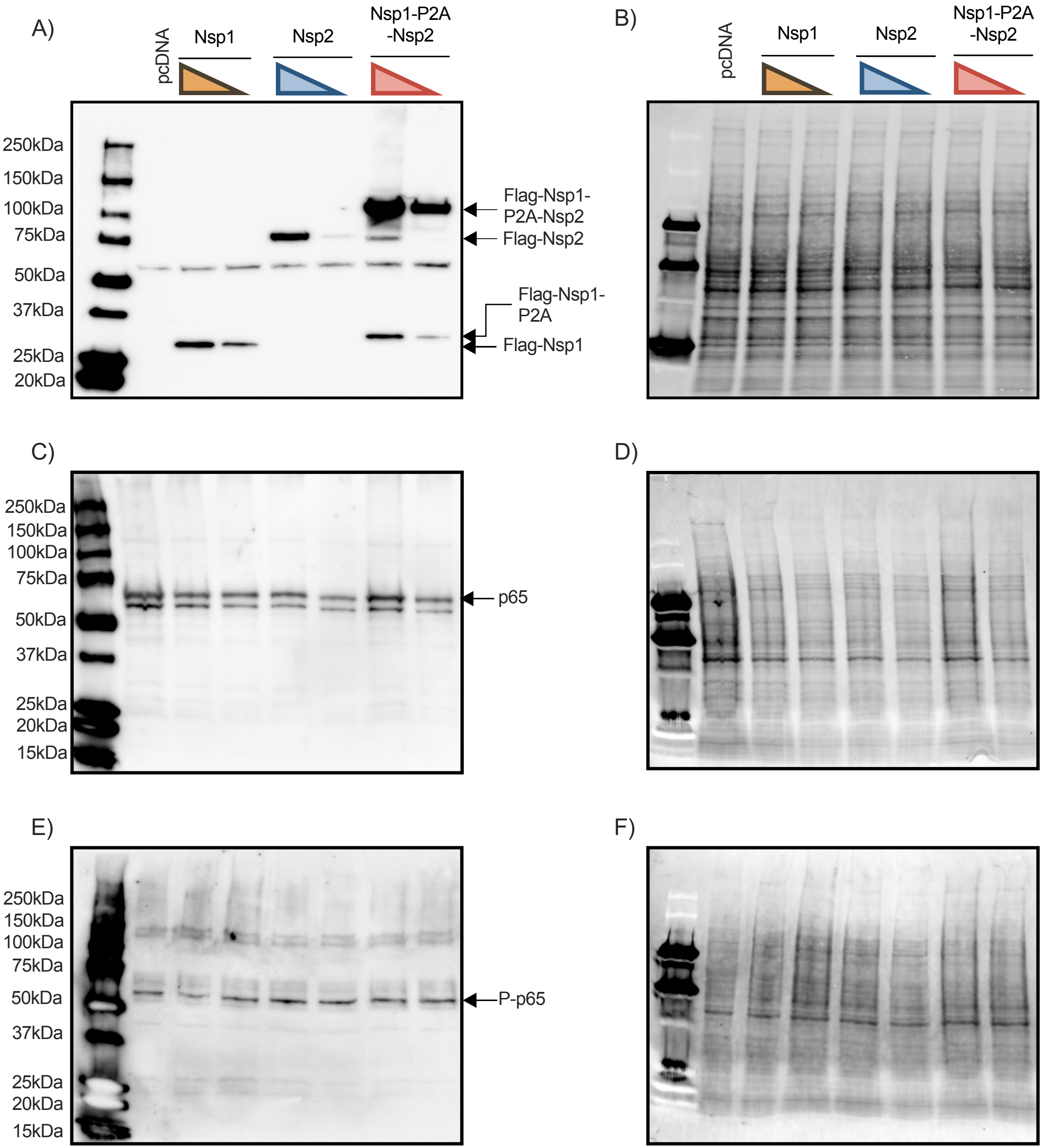

**Figure S2.** Uncropped blots (A, C, D) and Stain-Free blot (B, D, F) corresponding to the relative protein expression level of Nsp1 or Nsp2 (A, B), total p65 subunit (C,D) and phosphorylated p65 (E,F) following transfection with Nsp1, Nsp2 or polyprotein expression vector.

**Table S1.** Primers used for plasmid construction.

| **Plasmid construction** | Forward (5'-3') | Reverse (5'-3') |
|---|---|---|
| pENTR_SARS-CoV-2_Nsp1 | CACCATGGGAACCAATTCAGGAGAGCCTTGTCCCTGGTTTC | GGTCTAGATATCTCGAGTGCTTACCCTCCGTTAAGCTCAC |
| pENTR_SARS-CoV-2_Nsp2 | CACCATGGGAACCAATTCAGGCATACACTCGCTATGTC | GGTCTAGATATCTCGAGTGCTTAACCGCCTTTGAGTGTG |
| pCDNA5-Nsp1-P2A-Nsp2 | GATACATATGATCATGACATCGATTACAAGGATGACGATG | GTCATCGTCATCCTTGTAATCTGCTGCAGGACCTGGATTCT<br>CCTCCACATCTCCTGCCTGCTTTAACAGAGAGAAATTAGTA<br>GCGTATGCCCCTCCGTTAAGCTCACGC |
| pMAGIC-MYC-Nsp2(C.O) | TTTCTATACAAAGTTGCCACATGGAACAAAAACTCATCTCAGAAGAGGATCTGAAGCTTATGGCTTACACCCGCTAC | CTTTATTATACAAAGTTGCGTTAACCTCCCTTCAGGGTG |

**Table S2.** Primers and probes used for RT-qPCR and ddPCR experimentations.

| Mouse RT-qPCR and ddPCR | | | |
|---|---|---|---|
| | Forward (5'-3') | Reverse (5'-3') | Probe (5'-3') |
| *Cxcl1* | CCCAAACCGAAGTCATAGCC | TGGGGACACCTTTTAGCATC | - |
| *Ccl2* | AGAGCTACAAGAGGATCACC A | GTATGTCTGGACCCATTCCTTC | - |
| *Isg56* | CAGGATATTCACCTCCGCTAT G | CCTCCAAGCAAAGGACTTCT | - |
| *Ifny* | ATGAACGCTACACACTGCAT C | CCATCCTTTTGCCAGTTCCTC | - |
| *Ifnα* panel | GCAACCCTCCTAGACTCATTC TGC | TATKTCCTCACAGCCAGCAG | - |
| *Gapdh* | AACTTTGGCATTGTAGAAGG | ACACATTGGGGTTAGGAACA | - |
| *Rpp30* | AGATTTGGATTTAAGAGCG | GAGCAGCAGTCTCCACGAGT | 5HEX/AGAGCCTTC/ZEN/AGGTCTGAG CC/3IABkFQ |
| **Human RT-qPCR and ddPCR** | | | |
| | Forward (5'-3') | Reverse (5'-3') | Probe (5'-3') |
| *IFNβ* | AAACTCATGAGCAGTCTGCA | AGGAGATCTTCAGTTTCCCAGG | 56-FAM/ATGGTCCAGGCACAGTGACTGT CCTC/3BHQ_1 |
| *GAPDH* | TTCACACCCATGACGAACAT | AATCCCATCACCATCATCTTCCAG | 5HEX/CGACGTACT/ZEN/CAGCGCCA GCATC/3IABkFQ |
| *ISG15* | CATGGGCTGGGACCTGACG | CGCCAATCTTCTGGGTGATCTG | - |
| *ISG56* | CTTGAGCCTCCTTGGGTTCG | GCTGATATCTGGGTGCCTAAGG | - |
| SARS-CoV-2_E | TTCTTGCTTTCGTGGTATTCT | GACTCACGTTAACAATATTGCAG | E_SARBECO_P1 PROBE |

**Table S3.** Genes included in RT$^2$ profiler PCR Arrays Mouse Antiviral Response (PAMM-122ZR).

| Symbol | Description | Symbol | Description |
|---|---|---|---|
| Aim2 | Absent in melanoma 2 | Map2k1 | Mitogen-activated protein kinase kinase 1 |
| Atg12 | Autophagy-related 12 (yeast) | Map2k3 | Mitogen-activated protein kinase kinase 3 |
| Atg5 | Autophagy-related 5 (yeast) | Map3k1 | Mitogen-activated protein kinase kinase kinase 1 |
| Azi2 | 5-azacytidine induced gene 2 | Map3k7 | Mitogen-activated protein kinase kinase kinase 7 |
| Card9 | Caspase recruitment domain family, member 9 | Mapk1 | Mitogen-activated protein kinase 1 |
| Casp1 | Caspase 1 | Mapk14 | Mitogen-activated protein kinase 14 |
| Casp8 | Caspase 8 | Mapk3 | Mitogen-activated protein kinase 3 |
| Ccl3 | Chemokine (C-C motif) ligand 3 | Mapk8 | Mitogen-activated protein kinase 8 |
| Ccl4 | Chemokine (C-C motif) ligand 4 | Mavs | Mitochondrial antiviral signaling protein |
| Ccl5 | Chemokine (C-C motif) ligand 5 | Mefv | Mediterranean fever |
| Cd40 | CD40 antigen | Mx1 | Myxovirus (influenza virus) resistance 1 |
| Cd80 | CD80 antigen | Myd88 | Myeloid differentiation primary response gene 88 |
| Cd86 | CD86 antigen | Nfkb1 | Nuclear factor of kappa light polypeptide gene enhancer in B-cells 1, p105 |
| Chuk | Conserved helix-loop-helix ubiquitous kinase | Nfkbia | Nuclear factor of kappa light polypeptide gene enhancer in B-cells inhibitor, alpha |
| Cnpy3 | Canopy 3 homolog (zebrafish) | Nlrp3 | NLR family, pyrin domain containing 3 |
| Ctsb | Cathepsin B | Nod2 | Nucleotide-binding oligomerization domain containing 2 |
| Ctsl | Cathepsin L | Oas2 | 2'-5' oligoadenylate synthetase 2 |
| Ctss | Cathepsin S | Pin1 | Protein (peptidyl-prolyl cis/trans isomerase) NIMA-interacting 1 |
| Cxcl10 | Chemokine (C-X-C motif) ligand 10 | Pstpip1 | Proline-serine-threonine phosphatase-interacting protein 1 |
| Cxcl11 | Chemokine (C-X-C motif) ligand 11 | Pycard | PYD and CARD domain containing |
| Cxcl9 | Chemokine (C-X-C motif) ligand 9 | Rela | V-rel reticuloendotheliosis viral oncogene homolog A (avian) |
| Cyld | Cylindromatosis (turban tumor syndrome) | Ripk1 | Receptor (TNFRSF)-interacting serine-threonine kinase 1 |
| Dak | Dihydroxyacetone kinase 2 homolog (yeast) | Spp1 | Secreted phosphoprotein 1 |
| Ddx3x | DEAD/H (Asp-Glu-Ala-Asp/His) box polypeptide 3, X-linked | Stat1 | Signal transducer and activator of transcription 1 |
| Ddx58 | DEAD (Asp-Glu-Ala-Asp) box polypeptide 58 | Sugt1 | SGT1, suppressor of G2 allele of SKP1 (S. cerevisiae) |
| Dhx58 | DEXH (Asp-Glu-X-His) box polypeptide 58 | Tank | TRAF family member-associated Nf-kappa B activator |
| Fadd | Fas (TNFRSF6)-associated via death domain | Tbk1 | TANK-binding kinase 1 |
| Fos | FBJ osteosarcoma oncogene | Tbkbp1 | TBK1 binding protein 1 |
| Hsp90aa1 | Heat shock protein 90, alpha (cytosolic), class A member 1 | Ticam1 | Toll-like receptor adaptor molecule 1 |
| Ifih1 | Interferon induced with helicase C domain 1 | Tlr3 | Toll-like receptor 3 |
| Ifna2 | Interferon alpha 2 | Tlr7 | Toll-like receptor 7 |
| Ifnar1 | Interferon (alpha and beta) receptor 1 | Tlr8 | Toll-like receptor 8 |
| Ifnb1 | Interferon beta 1, fibroblast | Tlr9 | Toll-like receptor 9 |
| Ikbkb | Inhibitor of kappaB kinase beta | Tnf | Tumor necrosis factor |
| Il12a | Interleukin 12A | Tradd | TNFRSF1A-associated via death domain |
| Il12b | Interleukin 12B | Traf3 | Tnf receptor-associated factor 3 |
| Il15 | Interleukin 15 | Traf6 | Tnf receptor-associated factor 6 |
| Il18 | Interleukin 18 | Trim25 | Tripartite motif-containing 25 |
| Il1b | Interleukin 1 beta | Actb | Actin, beta |
| Il6 | Interleukin 6 | B2m | Beta-2 microglobulin |
| Irak1 | Interleukin-1 receptor-associated kinase 1 | Gapdh | Glyceraldehyde-3-phosphate dehydrogenase |
| Irf3 | Interferon regulatory factor 3 | Gusb | Glucuronidase, beta |
| Irf5 | Interferon regulatory factor 5 | Hsp90ab1 | Heat shock protein 90 alpha (cytosolic), class B member 1 |
| Irf7 | Interferon regulatory factor 7 | MGDC | Mouse Genomic DNA Contamination |
| Isg15 | ISG15 ubiquitin-like modifier | RTC | Reverse Transcription Control |
| Jun | Jun oncogene | PPC | Positive PCR Control |
